# Cognitive Decline following Radiotherapy of Head and Neck Cancer: Systematic Review and Meta-Analysis of MRI Correlates

**DOI:** 10.3390/cancers13246191

**Published:** 2021-12-08

**Authors:** Noor Shatirah Voon, Hanani Abdul Manan, Noorazrul Yahya

**Affiliations:** 1 Diagnostic Imaging and Radiotherapy, Faculty of Health Sciences, National University of Malaysia, Jalan Raja Muda Aziz, Kuala Lumpur 50300, Malaysia; p98932@siswa.ukm.edu.my; 2 Functional Image Processing Laboratory, Department of Radiology, Universiti Kebangsaan Malaysia Medical Centre, Cheras, Kuala Lumpur 56000, Malaysia; hanani@ukm.edu.my

**Keywords:** neurocognition, head and neck cancer, magnetic resonance imaging, radiotherapy

## Abstract

**Simple Summary:**

Patients with nasopharyngeal carcinoma (NPC) following radiotherapy may show decline in their cognitive abilities. Early detection is essential for accurate treatment and prevention of cognitive decline. Our review is the first meta-analysis on the correlates between cognitive impairment and brain structural and functional changes. The review also showed that neuropsychological tests together with magnetic resonance imaging biomarkers have the potential to predict and monitor cognitive decline. In addition, cognitive decline following radiotherapy of head and neck cancer is dose-dependent with changes in brain imaging.

**Abstract:**

Radiotherapy for head and neck cancers exposes small parts of the brain to radiation, resulting in radiation-induced changes in cerebral tissue. In this review, we determine the correlation between cognitive deterioration in patients with head and neck cancer after radiotherapy and magnetic resonance imaging (MRI) changes. Systematic searches were performed in PubMed, Scopus, and Cochrane databases in February 2021. Studies of head and neck cancer patients treated with radiotherapy and periodical cognitive and MRI assessments were included. Meta-analysis was performed to analyse the correlation of Montreal Cognitive Assessment (MoCA) scores to MRI structural and functional changes. Seven studies with a total of 404 subjects (irradiated head and neck patients, *n* = 344; healthy control, *n* = 60) were included. Most studies showed the significance of MRI in detecting microstructural and functional changes in association with neurocognitive function. The changes were seen in various brain areas, predominantly the temporal region, which also shows dose dependency (6/7 studies). An effect size (r = 0.43, *p* < 0.001) was reported on the correlation of MoCA scores to MRI structural and functional changes with significant correlations shown among patients treated with head and neck radiotherapy. However, the effect size appears modest.

## 1. Introduction

Radiotherapy (RT) with or without the combination of chemotherapy is the primary treatment option for head and neck cancer patients [1,2,3]. During radiation, head and neck cancer (HNC) patients were irradiated with high doses of radiation to the tumour, with normal brain tissues and sometimes crucial brain components such as temporal lobes, the brain stem and the hippocampus, are within or in close proximity to the target volume [4,5,6,7,8]. The injury to these brain compartments may increase the risk of compromised intelligence, memory impairment, and learning disabilities that would negatively impact patients’ quality of life, including diminishing work productivity, reduced engagement in social activities, and difficulties in daily living [9,10,11,12]. In addition, radiation-induced brain injury was observed in long term survivors of small cell lung carcinoma, low-grade glioma, non-parenchymal tumours, primary brain tumours, nasopharyngeal cancer, and metastatic brain tumours treated with fractionated partial or whole brain irradiation showed deficits in both anatomic and functional components [13]. Intensity--modulated radiation therapy (IMRT) was shown to reduce long-term morbidity among head and neck patients, but long-term toxicities and quality of life (QoL) impairment remained considerable with hearing toxicity, hypothyroidism, depression, fatigue, and anxiety as some of the common adverse effects [14]. The development of late radiation toxicities such as cranial neuropathies and cognitive impairment years after treatment can also induce a significant decline in QoL [15].

Nevertheless, the improved precision of radiotherapy technology has successfully reduced radiation doses in normal brain tissues, thus reducing the risk of brain tissue necrosis in patients following radiotherapy treatment [6]. Because radiation-induced brain injury at acute and early delayed periods within the first six months after radiotherapy is often not detected by routine imaging [16,17], it could potentially cause cognitive decline and exert a permanent effect [18,19]. Currently, the long-term cognitive dysfunction yielded by the incidental radiation dose to the surrounding cerebral tissues is subject to active investigations by researchers across the globe [20,21,22,23]. To monitor the cognitive functions, several neurocognitive tests can be conducted, including Montreal Cognitive Assessments (MoCA), Mini-Mental State Exam, Auditory Verbal Learning Test (AVLT), Trail Making Test A, Trail Making Test B, Rey Auditory Verbal Learning Test, Wechsler Adult Intelligence Scale, and Hopkins Verbal Learning Test. However, studies showed lower Montreal Cognitive Assessments (MoCA) scores in patients following RT even without overt cerebral injury [6,24].

In recent years, advances in brain imaging such as fibre tracking by diffusion imaging and functional mapping have shown their potential in the radiosurgical and surgical management of brain tumours [25,26,27]. These advancements could be beneficial should they be employed in the management of head and neck cancer treatment by monitoring brain microstructural changes. Functional connectivity (FC) in functional magnetic resonance imaging (fMRI) studies had been explored in measuring the correlation of synchronised signal among spatially distributed brain regions with the deduction of regions with correlated activity from functional networks [28]. In addition, functional connectivity alterations may provide valuable information on functional recovery and treatment strategies, allowing the precision of dose distribution, reliable dose constraints, and prevention or minimisation of brain damage [29]. Diffusion imaging has also shown potential as an early non-invasive indicator in predicting the early response of radiotherapy-induced white matter damage in nasopharyngeal carcinoma (NPC) [30,31]. Thus, this may provide a potential biomarker for early intervention of cognitive impairments in patients [32,33,34,35].

Ideally, these biomarkers can be traced and monitored during treatment and follow-up evaluation with cognitive assessments to evaluate clinical outcome measures. However, studies on the early stages of impairment are scarce, with much focus on the late delayed effects of radiation treatment [19,20,21,22,23,24,25,26,27,28,29]. It is essential to understand that cognitive impairment occurs almost immediately following radiotherapy treatment and proper monitoring is necessary [36,37]. In this review, we summarise, through a systematic review, the role of neurocognitive and MRI assessments in monitoring the cognitive decline in head and neck patients. We also performed a meta-analysis of the correlation between cognitive assessment scores and MRI structural and functional changes.

## 2. Materials and Methods

### 2.1. Systematic Review Protocol

**Figure 1:**
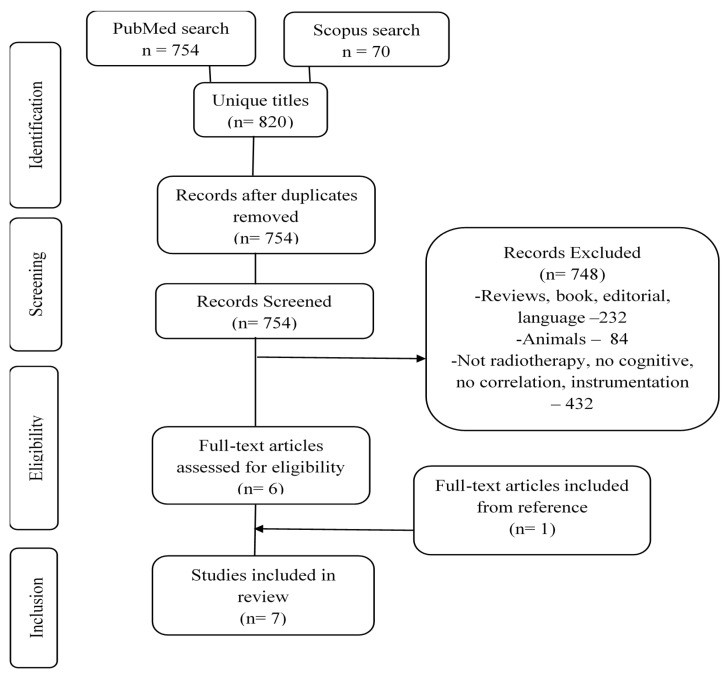
Search strategy via Preferred Reporting Items for Systematic Reviews and meta-analyses guidelines.

PubMed, Scopus, and Cochrane databases were utilised to search for relevant articles, published between the earliest record and 28 February 2021. The Preferred Reporting Items for Systematic Reviews and Meta-Analyses (PRISMA) was used as the reporting guidelines (Supplementary Table S1). No ethical approval was required. The search terms used for each database can be found in Supplementary Table S2 focusing on cognition, head and neck cancer, and radiation therapy. Manuscripts were evaluated for eligibility using the PICOS strategy (Supplementary Table S3) All full-text articles and original research manuscripts (i.e., not systematic reviews or meta-analyses) that investigated the neurocognitive changes in head and neck cancer patients following radiotherapy using cognitive and MRI assessments were included in this systematic review. Studies without the association of MRI changes to cognitive differences were not included in the current study. Animal studies or articles in a language other than English and Malay were not included. In the initial phase, manuscripts were reviewed to exclude articles not fulfilling the PICOS criteria via title alone, abstract, and full-text screening by NSV and NY independently. A mutual discussion by NSV and NY obviated the conflicts. In the second phase, to find all eligible articles, the references of the included articles were extracted and reviewed for additional studies. Spreadsheet software was used to organise and assess the titles of included studies and identify duplicate entries. Discrepancies in the results of the selection were deliberated in team meetings. Study search and selection were completed on 28 March 2021. The study review protocol is not published, with minor changes being made by adding meta-analysis. The detailed search strategy is illustrated in Figure 1.

### 2.2. Data Review and Extraction

Upon finalisation of the selection process, data extraction was performed by NSV and independently reviewed by NY. Information was extracted and the following data were included: manuscript title, authors, publication date, number of patients, cognitive assessment, magnetic resonance imaging (MRI) changes, and neurocognitive outcome. These are available in a private repository which can be shared upon request. The relationship between cognitive outcome to MRI and treatment dose will be the focus of this review.

### 2.3. Meta-Analysis

Meta-analysis was performed with studies having common region-of-interest, cognitive tests, and MRI changes. Analysis was performed using meta-essentials [38]. The relationship between MRI structural and functional differences and cognitive changes among studies evaluated will be the focus of this study. Analysis was done by selecting the highest correlation value of cognitive scores to MRI changes in each evaluated study and the number of subjects. The *p*-value reported was two-tailed, with *p* < 0.05 considered as significant. The effect size of the studies was generated. Publication bias was done to test if there are biases that can impact the study conclusions.

## 3. Results

### 3.1. Study Selection and Quality

A total of 820 records were produced from PubMed and Scopus databases. After removing duplicates, 754 articles were reviewed for inclusion with screening on the title, abstract, and full text based on the PICOS criteria, and six met the inclusion criteria. At the second stage, 330 references and citations of the selected articles were reviewed, and one additional studies was selected. Only studies involving nasopharyngeal carcinoma (NPC) fulfilled the inclusion criteria though the study aimed to evaluate cognitive changes in head and neck cancer patients corresponding with the search term used for database search. A detailed flow chart on the article selection process is illustrated in Figure 1.

Studies were assessed using the Quality Assessment Tool of Case-Control Studies or Quality Assessment Tool for Observational Cohort and Cross-Sectional Studies (Supplementary Table S4) to ensure studies are high in quality. Studies on the cognitive changes in head and neck cancer have only recently gained traction, probably due to the clinical interest in reducing cognitive deterioration following treatment; thus, a low sample size was realised. In this study, all brain changes, either structural, functional, or volumetric, were included. In total, three case-control longitudinal and four prospective cross-sectional studies were selected for analysis.

### 3.2. Study Characteristics

The publication date ranges from 2016 to 2020 with the summarised characteristics of the selected studies presented in Table 1.

A total of 344 patients were irradiated for head and neck cancer. All studies had patients irradiated with intensity-modulated radiotherapy (IMRT) except for one study that also incorporated tomotherapy as a mode of treatment [41]. Patient age ranged between 20 and 71 (median 49). Three studies were categorised as case-control longitudinal studies [39,40,41,43] with investigation done as early as one day after radiotherapy completion to 6 months later and four as prospective cross-sectional studies [29,36,37,42]. Several types of imaging investigation were done, such as functional magnetic resonance imaging (fMRI), diffusion kurtosis imaging (DKI) and three-dimensional brain volume imaging (3D-BRAVO) in monitoring patients brain structure with imaging done at 1.5 and 3.0 Tesla. A battery of neurocognitive tests was performed across the studies, with the most common was Montreal Cognitive Assessment (MoCA), which was utilised in all seven studies.

### 3.3. Neurocognitive Assessments in Detecting Cognitive Changes

Two neurocognitive tests were used in the studies evaluated, i.e., MoCA and AVLT [39], to detect cognitive changes following radiation treatment in head and neck cancer. Due to the low number of studies utilising other cognitive assessments, the present review will focus on MoCA, enabling better interpretation and summary. The MoCA is a rapid screening instrument for mild cognitive dysfunction that assesses various cognitive domains: attention and concentration, executive functions, memory, language, visuo-constructional skills, conceptual thinking, calculations, and orientation. It is a brief 30-question test that takes about 10 to 12 min to administer with a scoring range from zero to 30. The average and range of MoCA scores for the studies are reported in Table 2. From the findings, most studies reported a MoCA score <26 to define cognitive impairment. Only one study [37] adjusted for patient’s education and age. All studies also reported changes in the post-MRI findings corresponding to lower MoCA scores post-RT (Table 2).

### 3.4. Relationship of Neurocognitive Assessments to Magnetic Resonance Imaging (MRI)

According to Ma et al. [36], five functional connections were significantly correlated with MoCA overall scores with the attention domain also being significantly correlated to functional connectivity between vermis and hippocampus (r = 0.43, *p* < 0.001). This relationship was further explored by Ma et al. [42] and it was found that altered cerebellar-cerebral FCs were also significantly correlated to MoCA and attention scores, one of the seven subscores in MoCA, although results obtained were negatively correlated. From the findings, the change of correlation from negative to positive may implicate that the RT process might have impaired the anticorrelation between the two networks of NPC patients. The impaired anticorrelation between the dorsal attention and default networks may suggest deficits in cognitive and attention processing of NPC patients after RT [41]. In contrast, no correlation was found between network-level functional connectivity and cognition in Qiu et al. [29] (Table 3), although significantly reduced FC (*p* < 0.005) in the left anterior cingulate cortex (ACC), the right insular and bilateral executive control network (ECN) to MoCA scores three months post-RT were reported compared to pre-RT. 

No significant correlations between FC and MoCA tests and no significant changes in MoCA scores between pre-and post-RT and healthy controls were shown in Ren et al. [39]. Nevertheless, seven weeks post-RT, FC was significantly reduced in several cortical regions of DMN, including the precuneus, posterior cingulate cortex, medial prefrontal cortex, and other regions such as parahippocampus, cuneus, lingual gyrus, fusiform gyri, and calcarine sulcus [39]. Significant reduction in connectivity was also shown in post-RT patients compared to controls in multiple cerebellar-cerebral regions including the cerebellum, parahippocampal gyrus, hippocampus, fusiform gyrus, inferior frontal gyrus, inferior occipital gyrus, precuneus, and cingulate cortex [39].

In terms of volume, a significant negative correlation was reported between the reduced MoCA scores and expansion of ventricles [40] (Table 3). Significant volume losses in the bilateral hippocampus, bilateral GCL, left subiculum (SUB), and the right molecular layer was correlated with rapid cognitive function decline [41]. According to Wu et al. [37], Kurtosis mean-1 of white matter could predict late delayed neurocognitive impairment through changes in MoCA scores post-RT with the sensitivity of 84.2% and specificity of 87.5% in the receiver operating (ROC) curve. However, the study done by Sharma et al. [44] displayed voluminous, diffuse, radiation-induced white matter hyperintensities; changes were not associated with any neurocognitive assessments.

### 3.5. Effect of Radiotherapy Treatment Dose to Brain Structural and Functional Changes

According to the studies, brain structure and function changes were apparent in patients treated with NPC radiotherapy compared to untreated patients or healthy controls. This was reported in Ma et al. [36] (Table 4) with changes of the cerebellum, sensorimotor and cingulo-opecular FC shown in irradiated patients with altered cerebral-cerebral FCs within dorsal attention, frontal-parietal [42], and default-mode networks [39,42].

The findings showed that greater FC corresponded to lower MoCA and attention scores. Furthermore, the medial frontal gyrus within the default-mode networks is considered to be associated with executive function and decision making, which propagates information for higher-level processing response [36,42]. Abnormal connection of sensorimotor and cingulo-opercular networks with cerebellum shown might also imply the radiation-induced motor deficits and cognitive function abnormalities, especially attention changes [36]. Significant reduction in bilateral temporal lobe volume after RT [40] and differences in its white and grey matters among the neurocognitive function decline (NFD) group [37] suggests the dependency of changes in brain microstructure on radiation dose. Negative correlations were also displayed between the volume of the bilateral hippocampus, bilateral granule cell layer (GCL), and right middle lobe (ML) to mean dose of the ipsilateral hippocampus [41] and maximum irradiation dose of the right temporal lobe to the right insular FC within the salience network [29] (Table 4). No studies reported on the normal tissue compilation probability (NTCP) modelling for the temporal lobe.

### 3.6. Meta-Analysis on the Correlation of MoCA Scores to Brain Changes

**Figure 2:**
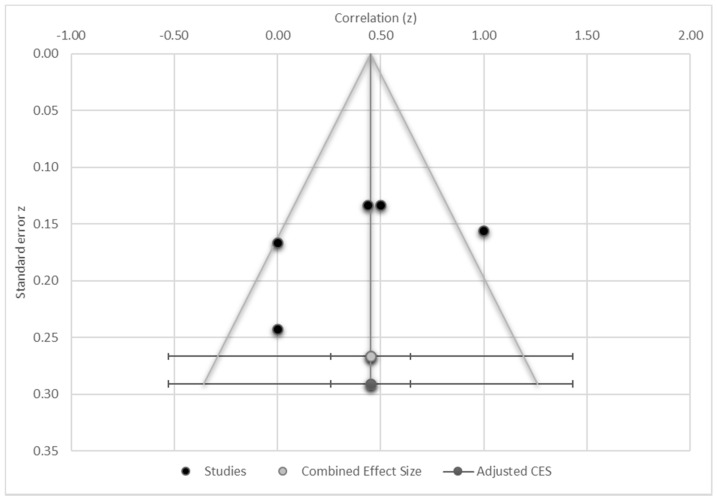
Funnel plot.

Analysis on the correlation of MoCA scores to MRI structural and functional changes was significantly heterogeneous (Q statistics = 23.10, I2 = 82.68%, *p* < 0.001) and statistically significant (Z = 2.21, *p* = 0.027) with a moderate effect size (r = 0.40) (Figure 2). The results indicate a significant moderate correlation between the cognitive and MRI assessments in head and neck cancers studies. Only two studies reported the attention sub score, which is inadequate to conduct a meta-analysis.

### 3.7. Publication Bias

Trim and Fill was used to investigate potential publication bias for the meta-analysed studies [45]. The adjusted estimate is found close to the original—in this setting, a correlation of 0.45 and the observed correlation of 0.45. Visually, minor asymmetry (observed and imputed studies) was shown with no evidence of publication bias in Egger’s test (*p* = 0.48), thus, publication bias is not a significant concern. Rosenthal’s Failsafe—N is 61 and Orwin’s Failsafe—N is 71, suggesting a need for approximately 70 studies with a mean effect size of 0.5 added to the analysis before the cumulative effect would become statistically non-significant.

## 4. Discussion

The results show a correlation between neurocognitive assessment scores to structural or functional changes following head and neck cancer radiotherapy. However, two studies found significant changes in several brain network functional connections (FC) in patients following radiotherapy, although the association with MoCA was absent [29,39]. Second, dose-dependent changes were also observed in the studies examined between pre-RT and healthy control to post-RT patients in the temporal region, predominantly the hippocampal region. Finally, our results exhibit good evidence on the potential of cognitive assessment with advanced MRI examination following the cognitive decline in head and neck radiotherapy to be introduced as a routine procedure in monitoring early brain changes.

All the studies indicate a cut-off point of 26 in MoCA assessments to define cognitive impairments. Though changes in MoCA scores were associated with MRI outcomes, the cut-off may be too stringent and not optimal among minorities [46] and certain health condition populations [47]. Additionally, the cut-off is also too high for cognitively normal older adults, even those who are highly educated [48]. Nevertheless, the use of MoCA is shown to be efficient in screening for mild cognitive impairment among the Chinese population [49,50] with the Cantonese Chinese MoCA being a consistent and reliable instrument [51]. Therefore, it is crucial to use age [48], education [46,48], and race or ethnicity [46,52] in correcting the cut-off scores to avoid misdiagnosis of cognitive decline. A lower MoCA cut-off score 23/30 yielded an overall better diagnostic accuracy with a lower false positive rate and excellent sensitivity (96%) and specificity (95%), thus, is recommended as the new MoCA cut-off score [52,53].

Across the studies, the neurocognitive assessment has shown the likelihood to be associated with MRI outcome following head and neck cancer radiotherapy, especially for the temporal region. Focus is given to the region due to its proximity to the target volume and would inevitably be incorporated into the treatment field, which exceeds the tolerance limit. Changes in functional connectivity (FC) and brain volume were significantly correlated with MoCA scores in most studies [36,37,40,41]. From the findings, the change of correlation from negative to positive may implicate that the RT process might have impaired the anticorrelation between the two networks of NPC patients. The impaired anticorrelation between the dorsal attention and default networks may suggest deficits in cognitive and attention processing of NPC patients after RT [42]. The correlation may also be inferred to be due to the radiation-induced cognitive impairment of domains such as short-term memory, visual memory, language ability, attention, and executive function [36,42]. In terms of volume, longitudinal changes in MoCA scores were associated with the longitudinal changes in total grey matter and bilateral temporal and ventricular volumes. Lv et al. [41] found that higher volume losses in the bilateral hippocampus, bilateral GCL, left subiculum, and right molecular layer were associated with a greater cognitive function decline. Furthermore, longitudinal dilation of ventricles was also correlated with the reduction in cognition as assessed by MoCA [40]. This indicates the atrophy of the hippocampal and several subfields may affect cognitive impairment in patients following RT [41]. In addition, grey matter loss [54] and cognitive impairment [55] have been linked with ventricular dilation; thus, the dilation may potentially serve as a cognitive impairment indicator in the clinical setting [40].

Radiation-induced changes were also observed throughout the studies investigated. The early changes are closely related to vascular damage shown by vessel dilation, endothelial cells loss, nuclei enlargement, vessel wall thickening, increased vessel permeability, and decreased vessel density and length [19,56]. In this review, resultant functional connectivity and brain volumes from irradiation were observed in multiple cerebellar regions. This was shown with the altered correlation between brain networks observed in NPC patients following RT, which may imply deficits in cognitive and attention processing [36,42]. In addition, the demonstrated differences in the FC pattern also suggests that radiation-induced changes may not be bound to the exposed area only, but other encephalic regions such as the cerebellum, sensorimotor, and cingulo-opercular areas [36]. This shows that the incidental radiation received by the brain during treatment of HNC could contribute to cognitive impairment [57]. The findings suggest that early microstructural injury of the temporal lobe has a direct contributory relation to the delayed neurocognitive decline with lower MoCA scores shown post-radiotherapy [37]. Specifically, an increased radiation dose to the temporal lobes and cerebellum were significantly associated with worse memory performance and motor coordination, respectively [57]. In addition, a higher radiation dose (30 Gy) induced earlier and more severe histological changes than a lower dose that were reflected with changes in diffusivity and perfusion [58,59,60]. Nevertheless, in the study done by Zer et al. [61], no significant correlation was shown to suggest the risk of treatment parameters, such as chemotherapy regimen or radiation dose, to greater cognitive decline.

Radiation-induced atrophy was also demonstrated in the bilateral hippocampus, bilateral GCL, and right molecular layer [17,41,62], suggesting the atrophy of the subfields is primarily induced by radiation that might be associated with early radiation effects on vascular injury, reduced molecular layer volume, and disruption of neuronal structure and synaptic integrity [41]. The elevated volume losses in these areas were associated with a rapid cognitive function decline evaluated by MoCA in irradiated patients [41], indicating dose-dependent atrophy. Additionally, altered FC within the default-mode and salience networks also indicates high-order cognition impairment, especially memory and attention [29]. According to Wen et al. [63], limiting the dose delivered to 0.5-cm^3^ temporal lobe volume (D0.5cc) to less than 65.06 Gy may be advisable during IMRT for NPC patients, as it decreases the risk of temporal lobe injury (TLI) in older patients with advanced tumour stage. Thus, the implementation of NTCP modelling could potentially predict TLI and allow individualised follow-up management. Therefore, a clinically appropriate and safe dose is crucial in protecting these vulnerable regions.

From this review, a significant moderate correlation was shown between MoCA scores and MRI changes. As varied results were obtained from the studies, a meta-analysis conclusion was made based on the assumption of whole-brain analysis rather than region-specific. Significance in the correlation of MoCA score to functional connectivity or brain volume was shown in several studies [36,37,40,41,42]. This suggests the possibility of determining and monitoring the cognitive decline in patients following head and neck cancer by implementing neurocognitive assessment and MRI examinations as standard protocol. However, two of the evaluated studies [29,39] did not show any relationship between FC and cognition, which may be due to the short intervals following RT, two independent sample groups, and insensitivity of cognitive screening tool [64].

Several issues may restrict current analysis due to the diversity in the treatment regime, experimental parameters, and study groups. First, studies included two study designs: longitudinal and cross-sectional. However, changes in the cognitive and brain morphology in the cross-sectional studies may differ across patients. Thus, an accurate representation of the changes between studies could not be projected. Second, the neurocognitive assessment being used by the studies, MoCA is a brief cognitive screening tool, although being the most commonly used assessment. This is due to its easy accessibility and management in assessing the general cognitive function of head and neck patients, given that different cognitive domains were evaluated. Nevertheless, it is not sensitive to certain domains, such as verbal and visual memory, executive functions, and attention [13,65]. Third, almost all studies enrolled patients with concurrent chemotherapy and RT. Although no chemotherapy-related changes were observed from the studies [29,37,39,40,41] the synergy between chemotherapy and radiotherapy may affect the results. According to Gan et al. [57], chemoradiotherapy was associated with worse cognitive dysfunction, and specific agents such as methotrexate and 5-fluorouracil are believed to cause changes in diffusion imaging due to their neurotoxicity nature [66,67] in specific brain regions, including the frontal lobes and hippocampus [67]. Advancement in chemotherapy protocols with better implementation and higher intensification may change the effects. Therefore, a separate analysis is suggested for patients with RT only to elucidate the RT-specific effects. Fourth, as studies implemented various imaging protocols, cognitive domains, and endpoints, analyses provided may have varied between studies. However, from this review, the correlation between cognitive scores and MRI findings was shown. Fifth, as the study includes multi-modalities in concluding the correlation of cognitive scores with brain changes, summarisation in the results may differ between modalities. Nevertheless, the approach taken is reasonable as a diagnosis is often made with multi-modal imaging. Finally, confounding factors such as treatment interventions, disease progression, and functional deficit may influence cognition [68]. As patients were tested at different ages, the cognitive changes may be due to aging with declines in cognitive function abilities, declines in grey and white matter volume, changes in white matter integrity, and reductions in neurotransmitter levels [69]. Nevertheless, the correlation of cognitive abilities to the age of patients was not discussed as most of the studies did not consider age as a factor in their analysis. In addition, the different time points in post-radiotherapy assessment may also influence the cognitive outcomes as changes in brain structure were most severe within six months after radiotherapy, and different regions exhibited distinct recovery rates [30] with neurocognitive function progressively deteriorates after two years of treatment [61].

## 5. Conclusions

Implementation of neurocognitive assessment with advanced MRI examination in monitoring brain microstructural and functional changes of head and neck cancer patients could detect cognitive changes early. With suitable intervention, further deleterious effects on the patient’s cognition can be prevented. Thus, the inclusion of both assessments could improve patient’s care by preventing secondary damages that could occur following head and neck radiotherapy. However, further validation studies are required to better interpret and understand the association of neurocognitive assessment to brain structural and functional changes on specific cognitive domains and dose-dependent changes.

## Figures and Tables

**Table 1:** Study characteristics.

	First Author [ref.]	Intervention	Pts No	Median Age (years)	Group Division	Male (%)	Cancer (%)		Chemo- Therapy (%)	Imaging Investigation	Neurocognitive Test
							Site	Staging (AJCC)			
Longitudinal study											
1.	Ren WT (2019) [39]	IMRT	20 (NPC), 17 (NC)	46.3	NC, *n* = 20 NPC, *n* = 22 (baseline, 1 day after RT completion)	80	NPC, 100%	I/II, 29%; III/IV, 71%	60	rs-fMRI, FC	MoCA, AVLT
2.	Guo Z (2018) [40]	IMRT	63 (NPC), 20 (NC)	49 (21–62)	NC, *n* = 20 NPC, *n* = 63 (scan at pre and post RT (at 3 months or 6 months)	68	NPC, 100%	NA	93.7	3D-BRAVO	MoCA
3.	Lv X (2018) [41]	IMRT TOMO	58 (NPC): 53-IMRT; 5-Tomo, 20 (NC)	21–62	NC, *n* = 20 (baseline, 3–4 months and 6–7 months) NPC, pre RT, *n* = 58; post RT (3 months), *n* = 45; post RT (6 months), *n* = 32	67.2	NPC, 100%	I, 1.7%; II, 12%; III, 46.6%; IV,39.7	94.8	3D- BRAVO	MoCA
Prospective cross-sectional											
1.	Wu G (2020) [37]	IMRT	44 (NPC)	20–71	NFD, *n* = 16; NFND, *n* = 38	65.9	NPC, 100%	I-II, 53.7%; III-IV, 46.3%	94.4	DKI	MoCA
2.	Ma Q (2017) [42]	IMRT	59 (NPC)	20–55	Baseline NPC, *n* = 24; Complete RT NPC, *n* = 35	72.9	NPC, 100%	NA	100	fMRI, FC	MoCA
3.	Qiu Y (2017) [29]	IMRT	39 (NPC)	48.9 (22–63)	NPC, *n* = 39 (baseline, 3 months post RT)	64	NPC, 100%	II, 7.7%; III-IV, 92.3%	100	BOLD-fMRI, FC	MoCA
4.	Ma Q (2016) [36]	IMRT	59 (NPC)	20–55	Baseline NPC, *n* = 24; Complete RT NPC, *n* = 35	72.9	NPC, 100%	NA	100	fMRI, FC	MoCA

Notes: Abbreviations:NPC—nasopharyngeal carcinoma; NC—normal control; rsfMRI—resting state functional MRI; BOLD-fMRI—Blood oxygen level dependent-functional MRI; DKI—diffusion kurtosis imaging; IMRT—intensity-modulated RT; Tomo—tomotherapy; 3D-BRAVO—three dimensional brain volume imaging; MoCA—Montreal Cognitive Assessment; FC—functional connectivity; AVLT—auditory verbal learning test; NFD—neurocognitive function decline; NFND—neurocognitive non-function decline.

**Table 2:** MoCA scores and changes in MRI findings.

First Author, Year	Average MoCA Post-RT (Range, Bonus Point)	Pre-MRI Findings	Post-MRI Findings	Study Limitations
Ma Q, 2016 [36]	24.2 (22–27)		45 altered FC compared to untreated NPC group	Heterogeneous treatment protocol, combined both non-irradiated and irradiated subjects, varied sample size, lack of new and larger sample, and between-subject variance
Qiu Y, 2017 [29]	NR	Functional network connectivity for NPC patients pre- and post-RT shared similar connectivity	Weaker intra-network connectivity with lower mean connectivity correlation than baseline	Heterogeneous treatment protocols, between-subject variance
Ma Q, 2017 [42]	24.2 (22–27)		Altered FC between cerebellar seeds and relative brain clusters	Heterogeneous treatment protocol, combined both non-irradiated and irradiated subjects, varied sample size, lack of new and larger sample, and between-subject variance
Guo Z, 2018 [40]	<26	No differences in cerebral volume of pre-NPC to controls	Decrease in brain macrostructural volume	Combined both non-irradiated and irradiated subjects, short time interval, and varied sample size
Lv X, 2018 [41]	NR	No significant differences in volumes of hippocampus and hippocampal subfields between groups	Significant volume reductions in bilateral hippocampus and hippocampal subfields	Combined both non-irradiated and irradiated subjects and varied sample size
Ren WT, 2019 [39]	27 (24–29)	No significant changes in regional cerebral and connectivity before RT	Reduced regional cerebral and neural network functions	Comparison to healthy controls and small sample size, short time interval
Wu G, 2020 [37]	<26 (<12 years education and >65 years age)	Baseline of kurtosis and diffusivity does not show significant difference	Significantly lower kurtosis and diffusivity of white matter	Heterogeneous treatment protocols, comparison between different marker groups, and between subject-variance

**Table 3:** Relationship of neurocognitive outcome to MRI findings.

First Author, Year	Score	Functional Connectivity or Volume	Significant Relationships and Prediction Details	Summary
Functional connectivity				
Ma Q, 2016 [36]	MoCA	Vermis and hippocampus	r = 0.4440, *p* = 0.00043	↓ FC ↓ MoCA score
	Attention		r = 0.4282, *p* = 0.00072	↓ FC ↓ Attention score
	MoCA	Cerebellum lobule VI and dIPFC	r = −0.4343, *p* = 0.00059	↑ FC ↓ MoCA score
		Precuneus and dFC	r = 0.4622, *p* = 0.00023	↓ FC ↓ MoCA score
		Cuneus and middle occipital lobe	r = 0.4282, *p* = 0.00071	↓ FC ↓ MoCA score
		Anterior insula and cuneus	r = 0.4569, *p* = 0.00028	↓ FC ↓ MoCA score
Qiu Y, 2017 [29]	MoCA	Left anterior cingulate cortex within the default mode network (DMN)		No significant correlation
		Right insular within salience network (SN)		No significant correlation
		Bilateral executive control network (ECN)		No significant correlation
Ma Q, 2017 [42]	MoCA	Right cerebellar lobule VIIb and right fusiform gyrus	r = −0.34, *p* = 0.008	↑ FC ↓ MoCA score
	Attention		r = −0.41, *p* = 0.002	↑ FC ↓ Attention score
	MoCA	Left cerebellar lobule VIII and right crus I	r = −0.30, *p* = 0.021	↑ FC ↓ MoCA score
	Attention		r = −0.32, *p* = 0.001	↑ FC ↓ Attention score
	Attention	Left cerebellar lobule VIII and right MFG	r = −0.27, *p* = 0.040	↑ FC ↓ Attention score
Ren WT, 2019 [39]	MoCA	Default mode network (DMN)		No significant correlation
Volume				
Guo Z, 2018 [40]	MoCA	Ventricular	b_βvolume_ = −4.63 × 10^−4^, *p* = 0.007	↓ Volume ↓ MoCA score
Lv X, 2018 [41]	MoCA	Left hippocampus	b_βvolume_ = 0.010, *p* = 0.017	↓ Volume ↓ MoCA score
		Right Hippocampal	b_βvolume_ = 0.013, *p* = 0.002	↓ Volume ↓ MoCA score
		Left Subiculum	b_βvolume_ = 0.061, *p* = 0.018	↓ Volume ↓ MoCA score
		Left Granule cell layer (GCL)	b_βvolume_ = 0.102, *p* = 0.011	↓ Volume ↓ MoCA score
		Right Granule cell layer (GCL)	b_βvolume_ = 0.158, *p* = 0.022	↓ Volume ↓ MoCA score
		Right molecular layer (ML)	b_βvolume_ = 0.285, *p* = 0.002	↓ Volume ↓ MoCA score
Kurtosis				
Wu G, 2020 [37]	MoCA	Hippocampal	r = 0.76, *p* < 0.05	Kurtosis mean-1 best in predicting MoCA scores decline

**Table 4:** Dose-dependent changes with brain microstructure or functional connectivity.

First Author, Year	Dose-Dependent Changes
Ma Q, 2016 [36]	Functional connectivity pattern in NPC treated patients was significantly impaired compared to NPC untreated with changes shown in cerebellum, sensorimotor, and cingulo-opercular.
Qiu Y, 2017 [36]	Changes in right insular functional connectivity were negatively correlated with dose of right temporal lobe.
Ma Q, 2017 [42]	Altered cerebral-cerebral functional connectivity within dorsal attention, default, and frontoparietal networks shown in NPC treated patients.
Guo Z, 2018 [40]	Significantly decrease volume in bilateral temporal lobe with increased mean dose to this region.
Lv X, 2018 [41]	Volume deficits in the bilateral hippocampus, bilateral granule cell layer, and right molecular layer negatively correlates with the mean dose to ipsilateral hippocampus.
Ren WT, 2019 [39]	Decreased connectivity in multiple cerebellar-cerebellar regions mainly in the default-mode networks likely because of radiation dose.
Wu G, 2020 [37]	Significant radiation-induced changes in both white and gray matter of the temporal lobes due to the high radiation dose received.

## Data Availability

The data presented in this review are available in the main article or Supplementary Materials.

## Supplementary Materials

The following are available online at https://www.mdpi.com/article/10.3390/cancers13246191/s1: Table S1. PRISMA checklist; Table S2. Search strategy using Pubmed and Scopus databases; Table S3. PICOS criteria for inclusion; Table S4. Quality check. Reference [70] is referred to in Supplementary Materials.
