# Supplementary material for: Cognitive Decline following Radiotherapy of Head and Neck Cancer: Systematic Review and Meta-Analysis of MRI Correlates
Source: Cancers (Basel). 2021 Dec 8;13(24):6191. doi: 10.3390/cancers13246191 (PMC8699377; doi:10.3390/cancers13246191)
Supplement: Supplementary file 1 [file cancers-13-06191-s001.zip › cancers-1388573-supplementary.pdf]

Table S1. PRISMA Checklist.

Section and Topic	Item #	Checklist item	Location where item is reported
**TITLE**			**PAGE**
Title	1	Identify the report as a systematic review.	1
**ABSTRACT**			
Abstract	2	See the PRISMA 2020 for Abstracts checklist.	2
**INTRODUCTION**			
Rationale	3	Describe the rationale for the review in the context of existing knowledge.	4
Objectives	4	Provide an explicit statement of the objective(s) or question(s) the review addresses.	4
**METHODS**			
Eligibility criteria	5	Specify the inclusion and exclusion criteria for the review and how studies were grouped for the syntheses.	4
Informatio n sources	6	Specify all databases, registers, websites, organisations, reference lists and other sources searched or consulted to identify studies. Specify the date when each source was last searched or consulted.	4
Search strategy	7	Present the full search strategies for all databases, registers and websites, including any filters and limits used.	SUPPLEMEN TARY 2
Selection process	8	Specify the methods used to decide whether a study met the inclusion criteria of the review, including how many reviewers screened each record and each report retrieved, whether they worked independently, and if applicable, details of automation tools used in the process.	4
Data collection process	9	Specify the methods used to collect data from reports, including how many reviewers collected data from each report, whether they worked independently, any processes for obtaining or confirming data from study investigators, and if applicable, details of automation tools used in the process.	4
Data items	10a	List and define all outcomes for which data were sought. Specify whether all results that were compatible with each outcome domain in each study were sought (e.g. for all measures, time points, analyses), and if not, the methods used to decide which results to collect.	5
	10b	List and define all other variables for which data were sought (e.g. participant and intervention characteristics, funding sources). Describe any assumptions made about any missing or unclear information.	5
Study risk of bias assessment	11	Specify the methods used to assess risk of bias in the included studies, including details of the tool(s) used, how many reviewers assessed each study and whether they worked independently, and if applicable, details of automation tools used in the process.	5
Effect measures	12	Specify for each outcome the effect measure(s) (e.g. risk ratio, mean difference) used in the synthesis or presentation of results.	5
Synthesis methods	13a	Describe the processes used to decide which studies were eligible for each synthesis (e.g. tabulating the study intervention characteristics and comparing against the planned groups for each synthesis (item #5)).	SUPPLEMEN TARY 3
	13b	Describe any methods required to prepare the data for presentation or synthesis, such as handling of missing summary statistics, or data conversions.	5
	13c	Describe any methods used to tabulate or visually display results of individual studies and syntheses.	4
	13d	Describe any methods used to synthesize results and provide a rationale for the choice(s). If meta-analysis was performed, describe the model(s), method(s) to identify the presence and extent of statistical heterogeneity, and software package(s) used.	5
	13e	Describe any methods used to explore possible causes of heterogeneity among study results (e.g. subgroup analysis, meta-regression).	5
	13f	Describe any sensitivity analyses conducted to assess robustness of the synthesized results.	5
Reporting bias assessment	14	Describe any methods used to assess risk of bias due to missing results in a synthesis (arising from reporting biases).	5

Certainty assessment	15	Describe any methods used to assess certainty (or confidence) in the body of evidence for an outcome.	5

Section and Topic	Item #	Checklist item	Location where item is reported
**RESULTS**			**PAGE**
Study selection	16a	Describe the results of the search and selection process, from the number of records identified in the search to the number of studies included in the review, ideally using a flow diagram.	6
	16b	Cite studies that might appear to meet the inclusion criteria, but which were excluded, and explain why they were excluded.	7
Study characteristics	17	Cite each included study and present its characteristics.	6
Risk of bias in studies	18	Present assessments of risk of bias for each included study.	8
Results of individual studies	19	For all outcomes, present, for each study: (a) summary statistics for each group (where appropriate) and (b) an effect estimate and its precision (e.g. confidence/credible interval), ideally using structured tables or plots.	TABLE 1
Results of syntheses	20a	For each synthesis, briefly summarise the characteristics and risk of bias among contributing studies.	6-9
	20b	Present results of all statistical syntheses conducted. If meta-analysis was done, present for each the summary estimate and its precision (e.g. confidence/credible interval) and measures of statistical heterogeneity. If comparing groups, describe the direction of the effect.	8
	20c	Present results of all investigations of possible causes of heterogeneity among study results.	8
	20d	Present results of all sensitivity analyses conducted to assess the robustness of the synthesized results.	8
Reporting biases	21	Present assessments of risk of bias due to missing results (arising from reporting biases) for each synthesis assessed.	9
Certainty of evidence	22	Present assessments of certainty (or confidence) in the body of evidence for each outcome assessed.	8
**DISCUSSION**			
Discussion	23a	Provide a general interpretation of the results in the context of other evidence.	9
	23b	Discuss any limitations of the evidence included in the review.	10
	23c	Discuss any limitations of the review processes used.	10
	23d	Discuss implications of the results for practice, policy, and future research.	10
**OTHER INFORMATION**			
Registration and protocol	24a	Provide registration information for the review, including register name and registration number, or state that the review was not registered.	NA
	24b	Indicate where the review protocol can be accessed, or state that a protocol was not prepared.	5
	24c	Describe and explain any amendments to information provided at registration or in the protocol.	5
Support	25	Describe sources of financial or non-financial support for the review, and the role of the funders or sponsors in the review.	11
Competin g interests	26	Declare any competing interests of review authors.	11

Availability of data, code and other materials	27	Report which of the following are publicly available and where they can be found: template data collection forms; data extracted from included studies; data used for all analyses; analytic code; any other materials used in the review.	5

From: Page MJ, et al. 2020 [70]. For more information, visit: http://www.prisma-statement.org/ (accessed on 4 September 2021).

Table S2. Search strategy using Pubmed and Scopus databases

Database	Search string	Articles found
**Stage 1**		
Pubmed	(((("head and neck neoplasms"[MeSH Terms] OR ("head"[All Fields] AND "neck"[All Fields] AND "neoplasms"[All Fields]) OR "head and neck neoplasms"[All Fields] OR ("head"[All Fields] AND "neck"[All Fields] AND "cancer"[All Fields]) OR "head and neck cancer"[All Fields] OR ("oropharyngeal neoplasms"[MeSH Terms] OR ("oropharyngeal"[All Fields] AND "neoplasms"[All Fields]) OR "oropharyngeal neoplasms"[All Fields] OR ("oropharyngeal"[All Fields] AND "carcinoma"[All Fields]) OR "oropharyngeal carcinoma"[All Fields]) OR ("nasopharyngeal carcinoma"[MeSH Terms] OR ("nasopharyngeal"[All Fields] AND "carcinoma"[All Fields]) OR "nasopharyngeal carcinoma"[All Fields]) OR (("hypopharynx"[MeSH Terms] OR "hypopharynx"[All Fields] OR "hypopharyngeal"[All Fields]) AND ("carcinoma"[MeSH Terms] OR "carcinoma"[All Fields] OR "carcinomas"[All Fields] OR "carcinoma s"[All Fields])) OR ("salivary gland neoplasms"[MeSH Terms] OR ("salivary"[All Fields] AND "gland"[All Fields] AND "neoplasms"[All Fields]) OR "salivary gland neoplasms"[All Fields] OR ("salivary"[All Fields] AND "gland"[All Fields] AND "cancer"[All Fields]) OR "salivary gland cancer"[All Fields]) OR ("tongue neoplasms"[MeSH Terms] OR ("tongue"[All Fields] AND "neoplasms"[All Fields]) OR "tongue neoplasms"[All Fields] OR ("tongue"[All Fields] AND "cancer"[All Fields]) OR "tongue cancer"[All Fields]) OR ("mouth neoplasms"[MeSH Terms] OR ("mouth"[All Fields] AND "neoplasms"[All Fields]) OR "mouth neoplasms"[All Fields] OR ("oral"[All Fields] AND "cavity"[All Fields] AND "cancer"[All Fields]) OR "oral cavity cancer"[All Fields]) OR (("paranasal sinuses"[MeSH Terms] OR ("paranasal"[All Fields] AND "sinuses"[All Fields]) OR "paranasal sinuses"[All Fields]) AND	810

	("cancer s"[All Fields] OR "cancerated"[All Fields] OR "canceration"[All Fields] OR "cancerization"[All Fields] OR "cancerized"[All Fields] OR "cancerous"[All Fields] OR "neoplasms"[MeSH Terms] OR "neoplasms"[All Fields] OR "cancer"[All Fields] OR "cancers"[All Fields]))) AND ("neurocognition"[All Fields] OR "neurocognitive"[All Fields] OR "neurocognitively"[All Fields])) OR ("cognition"[MeSH Terms] OR "cognition"[All Fields] OR "cognitions"[All Fields] OR "cognitive"[All Fields] OR "cognitively"[All Fields] OR "cognitives"[All Fields]) OR ("memories"[All Fields] OR "memory"[MeSH Terms] OR "memory"[All Fields] OR "memory s"[All Fields]) OR (("intelligence"[MeSH Terms] OR "intelligence"[All Fields] OR "intelligences"[All Fields] OR "intelligent"[All Fields] OR "intelligently"[All Fields] OR "intelligibilities"[All Fields] OR "intelligibility"[All Fields] OR "intelligible"[All Fields]) AND ("quotient"[All Fields] OR "quotients"[All Fields])) OR ("attention"[MeSH Terms] OR "attention"[All Fields] OR "attentions"[All Fields] OR "attention s"[All Fields] OR "attentional"[All Fields] OR "attentive"[All Fields] OR "attentively"[All Fields] OR "attentiveness"[All Fields]) OR (("global"[All Fields] OR "globalism"[All Fields] OR "globalize"[All Fields] OR "globalized"[All Fields] OR "globalizes"[All Fields] OR "globalizing"[All Fields] OR "globally"[All Fields] OR "globals"[All Fields] OR "internationality"[MeSH Terms] OR "internationality"[All Fields] OR "globalization"[All Fields]) AND ("cognition"[MeSH Terms] OR "cognition"[All Fields] OR "cognitions"[All Fields] OR "cognitive"[All Fields] OR "cognitively"[All Fields] OR "cognitives"[All Fields])) OR ("language"[MeSH Terms] OR "language"[All Fields] OR "languages"[All Fields] OR "language s"[All Fields] OR "programming languages"[MeSH Terms] OR ("programming"[All Fields] AND "languages"[All Fields]) OR "programming languages"[All Fields]) OR ("executive function"[MeSH Terms] OR ("executive"[All Fields] AND "function"[All Fields]) OR	

	"executive function"[All Fields])) AND ("radiotherapy"[MeSH Terms] OR "radiotherapy"[All Fields] OR "radiotherapies"[All Fields] OR "radiotherapy"[MeSH Subheading] OR "radiotherapy s"[All Fields]) AND ("magnetic resonance imaging"[MeSH Terms] OR ("magnetic"[All Fields] AND "resonance"[All Fields] AND "imaging"[All Fields]) OR "magnetic resonance imaging"[All Fields]))	
Scopus	(TITLE-ABS-KEY ("cognitive" OR "cognition" OR "memory" OR "attention" OR "language") AND TITLE-ABS-KEY (radiotherapy OR "radiation therapy") AND TITLE-ABS-KEY ("diffusion tensor") AND TITLE-ABS-KEY (head and neck cancer)	70
	Total unique articles	754
**Stage 2**		
	Citations and references from articles selected in Stage 1	355
	Included	2

Table S3. PICOS criteria for inclusion

	Criteria
P - patient	adult human patients (age >20) treated with radiation therapy.
I - intervention	all types of external beam radiotherapy involving head and neck cancers.
C - comparison	Studies were excluded if they contained no cognitive assessment or magnetic resonance imaging.
O - outcome	cognitive changes must be measured by cognitive and magnetic resonance imaging assessments.
S – type of study	Randomised-controlled trials, cohort (prospective or retrospective). Exclude studies with no statistical comparisons (case study or case series) or reviews. Studies with less than 10 irradiated subjects are excluded.

Table S4. Quality check

**Quality Assessment Tool for Observational Cohort and Cross-Sectional Studies**	**Lv (2018)** [41].			**Guo (2018)** [40].			**Ren (2019)** [39].		
**Criteria**	**Yes**	**No**	**Other**	**Yes**	**No**	**Other**	**Yes**	**No**	**Other**
			**(CD, NR, NA)***			**(CD, NR, NA)***			**(CD, NR, NA)***
1. Was the research question or objective in this paper clearly stated?	x			x			x		
2. Was the study population clearly specified and defined?	x			x			x		
3. Was the participation rate of eligible persons at least 50%?	x			x			x		
4. Were all the subjects selected or recruited from the same or similar populations (including the same time period)? Were inclusion and exclusion criteria for being in the study prespecified and applied uniformly to all participants?	x			x			x		
5. Was a sample size justification, power description, or variance and effect estimates provided?		x			x			x	
6. For the analyses in this paper, were the exposure(s) of interest measured prior to the outcome(s) being measured?		x			x			x	
7. Was the timeframe sufficient so that one could reasonably expect to see an association between exposure and outcome if it existed?	x			x			x		
8. For exposures that can vary in amount or level, did the study examine different levels of the exposure as related to the outcome (e.g., categories of exposure, or exposure measured as continuous variable)?		x			x			x	
9. Were the exposure measures (independent variables) clearly defined, valid, reliable, and implemented consistently across all study participants?	x			x			x		
10. Was the exposure(s) assessed more than once over time?	x			x			x		
11. Were the outcome measures (dependent variables) clearly defined, valid, reliable, and implemented consistently across all study participants?	x			x			x		
12. Were the outcome assessors blinded to the exposure status of participants?		x			x			x	
13. Was loss to follow-up after baseline 20% or less?	x			x			x		

14. Were key potential confounding variables measured and adjusted statistically for their impact on the relationship between exposure(s) and outcome(s)?	x	x	x

**Quality Assessment of Case-Control Studies**	**Ma (2016)** [36].			**Qiu (2017)** [29]			**Ma (2017)** [42].			**Wu (2020)** [37].		
**Criteria**	**Yes**	**No**	**Other (CD, NR, NA)***	**Yes**	**No**	**Other (CD, NR, NA)***	**Yes**	**No**	**Other (CD, NR, NA)***	**Yes**	**No**	**Other (CD, NR, NA)***
1. Was the research question or objective in this paper clearly stated and appropriate?	x			x			x			x		
2. Was the study population clearly specified and defined?	x			x			x			x		
3. Did the authors include a sample size justification?		x			x			x			x	
4. Were controls selected or recruited from the same or similar population that gave rise to the cases (including the same timeframe)?	x			x			x			x		
5. Were the definitions, inclusion and exclusion criteria, algorithms or processes used to identify or select cases and controls valid, reliable, and implemented consistently across all study participants?	x			x			x			x		
6. Were the cases clearly defined and differentiated from controls?	x			x			x			x		
7. If less than 100 percent of eligible cases and/or controls were selected for the study, were the cases and/or controls randomly selected from those eligible?	x			x			x			x		
8. Was there use of concurrent controls?		x		x				x			x	
9. Were the investigators able to confirm that the exposure/risk occurred prior to the development of the condition or event that defined a participant as a case?	x			x			x			x		
10. Were the measures of exposure/risk clearly defined, valid, reliable, and implemented consistently (including the same time period) across all study participants?	x			x			x			x		
11. Were the assessors of exposure/risk blinded to the case or control status of participants?		x			x			x			x	
12. Were key potential confounding variables measured and adjusted statistically in the analyses? If matching was used, did the investigators account for matching during study analysis?	x			x			x			x		
